# Investigation of Genetic Modifiers of Copper Toxicosis in Labrador Retrievers

**DOI:** 10.3390/life10110266

**Published:** 2020-10-31

**Authors:** Xiaoyan Wu, Elise R. den Boer, Manon Vos-Loohuis, Frank G. van Steenbeek, Glen R. Monroe, Isaäc J. Nijman, Peter. A. J. Leegwater, Hille Fieten

**Affiliations:** 1 Department of Clinical Sciences, Faculty of Veterinary Medicine, Utrecht University, Yalelaan 108, 3584 CM Utrecht, The Netherlands; emilywu20130412@gmail.com (X.W.); e.r.denboer@uu.nl (E.R.d.B.); m.vos@uu.nl (M.V.-L.); f.g.vansteenbeek@uu.nl (F.G.v.S.); p.a.j.leegwater@uu.nl (P.A.J.L.); 2 Center for Molecular Medicine, University Medical Center Utrecht, Universiteitsweg 100, 3584 CG Utrecht, The Netherlands; g.monroe@umcutrecht.nl (G.R.M.); inijman@umcutrecht.nl (I.J.N.)

**Keywords:** Wilson disease, Menkes disease, dog, copper, ATP7A, ATP7B, Labrador retriever, copper toxicosis, RETN

## Abstract

Copper toxicosis is a complex genetic disorder in Labrador retrievers characterized by hepatic copper accumulation eventually leading to liver cirrhosis. The variation of hepatic copper levels in Labrador retrievers has been partly explained by mutations in *ATP7A* c.980C>T and *ATP7B* c.4358G>A. To further elucidate the genetic background of this disease, we used targeted Next Generation Sequencing (NGS) in a cohort of 95 Labrador retrievers to analyze 72 potential modifier genes for variations associated with hepatic copper levels. Variants associated with copper levels were subsequently evaluated in a replication cohort of 144 Labrador retrievers. A total of 44 variants in 25 different genes were identified, of which four showed significant association with copper levels. Of the four variants found associated with hepatic copper levels in the NGS cohort, one was validated in the replication cohort. The non-reference allele of the variant NC_006602.3.g.52434480C>T in *RETN* resulting in amino-acid change p.Leu7Phe was associated with decreased hepatic copper levels. In humans, resistin is associated with severity of non-alcoholic fatty liver disease, fibrosis, cirrhosis and mitochondrial dysfunction in hepatocytes. Further studies are needed to investigate the biological function of *RETN* p.Leu7Phe in the development of copper toxicosis in Labrador retrievers.

## 1. Introduction

Copper (Cu) is an essential trace element, with an important role in many cellular processes [1]. It can be present in two oxidation states Cu(I)/Cu^+^ (cuprous ion) and Cu(II)/Cu^2+^ (cupric ion), which provides the ability to act as a recipient or donor for electrons. Cu atoms are incorporated in a variety of proteins, where they are mainly involved in redox reactions [2]. In addition, copper is involved in cell metabolism pathways, like the mitochondrial respiratory chain and is essential for the cellular energy metabolism [3]. Because of its ability to form reactive oxygen species (ROS) which damage organic molecules, free copper is highly toxic [1]. Therefore, copper homeostasis is carefully regulated. Copper is absorbed in the small intestine and transported through the portal system to the liver. The liver plays a central role in copper homeostasis and is involved in copper-storage, re-distribution and excretion via the bile [2]. At the cellular level, maintenance of copper metabolism is regulated by several copper transporters and chaperones. In enterocytes, CTR1, CTR2 and DMT1 take up copper from the small intestine. Transportation of copper from the basolateral part of the enterocyte into the portal circulation is carried out by ATP7A. Copper enters the hepatocytes via CTR1. Intracellularly, copper is sequestered by metallothionein and glutathione after which copper chaperones COX17, CCS and ATOX1 shuttle copper to their respective intracellular target proteins CcO in the mitochondria, SOD1 in the cytoplasm and ATP7B in the trans-Golgi network (TGN) [4]. COMMD1 and ATP7B are involved in copper excretion into the bile. Under high intracellular copper conditions, ATP7B traffics into late endosomes/lysosomes, and COMMD1, as a complex with CCD22-CCDC93, binds to ATP7B and mediates ATP7B trafficking and plays a role in ATP7B stability and fidelity [4,5]. Additionally, ATP7B incorporates copper into apo-ceruloplasmin to form holo-ceruloplasmin in the TGN, which is subsequently excreted into the plasma [4]. Ceruloplasmin is a copper-carrying protein in the blood, which is also involved in iron metabolism. For a more in depth review on copper homeostasis we refer to the recent review of Linder [4].

The effects of copper-metabolism imbalance are illustrated by inherited disorders resulting from mutations in the genes encoding for copper transporters *ATP7A* and *ATP7B* in humans. The X-linked copper deficiency disorder Menkes disease, caused by mutations in *ATP7A,* is characterized by cerebral and cerebellar degeneration, connective tissue abnormalities, coarse hair and a failure to thrive [6,7]. The autosomal recessive copper accumulation disorder Wilson disease, caused by mutations in *ATP7B*, is characterized by copper accumulation in the liver and sometimes the brain, resulting in hepatic cirrhosis and neuronal degeneration. In Wilson disease, there is a wide variety in phenotypic presentation, even in patients with the same mutations in *ATP7B.* Several studies into possible modifier genes were executed and several candidate polymorphisms are thought to modify the clinical phenotype, including *COMMD1* [8], *ATOX1* [9], *PNPLA3* [10], *MTHFR* [11], *XIAP* [12], and *DMT1* [13].

A complex form of copper toxicosis, in which genetic and environmental factors are involved, has been reported in the Labrador retriever [14]. The hepatic copper accumulation identified in the liver of clinical and subclinical Labrador retrievers varies with values of just above 400 ppm dw up to above 2000 ppm dw in some individuals [15,16]. The hepatic copper accumulation induces chronic hepatitis and liver cirrhosis, and leads to clinical disease usually at middle age [15,16]. In our observation, two-thirds of the dogs that present with clinical signs attributable to copper associated-hepatitis are female, however population studies to confirm a female predisposition are currently lacking. The dietary intake of copper and zinc affects hepatic copper levels in Labrador retrievers [17]. In some dogs, hepatic copper levels can be decreased by low-copper, high-zinc diets, however there is a variable response between dogs, with some dogs (re)-accumulating copper despite being fed a copper restricted diet [18]. We previously reported the association of variants of ATP7A and ATP7B with hepatic copper levels in Labrador retrievers. The missense variant *ATP7B*:c.4358G>A resulting in ATP7B:p.Arg1453Gln was associated with increased hepatic copper levels, while the missense variant *ATP7A* c.980C>T resulting in ATP7A:p.Thr327Ile was associated with lower hepatic copper levels [14]. These variants account for only 12.5% of the genetic variability in the hepatic copper levels in the breed [14]. Therefore, we expect that there are variants in other genes that contribute to the variation in hepatic copper levels in these dogs. Recently, we excluded variants in *COMMD1* as possible modifiers for copper associated hepatitis in the Dobermann and Labrador retriever [19].

In 95 Labrador retrievers we investigated the exons and intron-exon boundaries of 72 genes known to be involved in copper metabolism and hepatic disease with the aim to identify variants with an association to hepatic copper levels. Associated variants were subsequently genotyped in a replication cohort of 144 Labrador retrievers. We identified a missense variant in the resistin gene (*RETN*) that was negatively associated with hepatic copper levels.

## 2. Results

### 2.1. Dogs

A total of 239 Labrador retrievers were included in this study. Quantitative copper scores were available for 176 dogs. The correlation between copper score and quantitative copper was 0.82 (*p*-value 2.2 × 10^−16^, 95% confidence interval [0.76–0.87]). Ninety-five Labrador retrievers were included in the targeted Next Generation Sequencing (NGS) cohort. This group consisted of 65 females and 30 males with a median age of 5.3 (0.9–13.5) years at the moment of liver biopsy. The median rubeanic acid (RA) score for copper was 2 (range 1–5). Non-reference allele frequencies for *ATP7B*:c.4373G>A and *ATP7A:980C>T* were 0.34 and 0.29, respectively. The replication cohort consisted of 144 Labrador retrievers, of which 97 were females and 47 were males. The median age at sample collection was 7.0 (0.9–12.7) years and the median copper score was 2 (range 1–5). Non-reference allele frequencies for *ATP7B*:c.4373G>A and *ATP7A:c.980C>T* were 0.32 and 0.28 respectively. No significant differences in the baseline cohort characteristics *ATP7A* and *ATP7B* allele frequencies, female:male ratio and copper score were found between the NGS and replication cohort. Cohort characteristics are shown in Table 1. Univariate analysis of the correlation of age at biopsy and hepatic copper levels did not show a significant correlation (*p*-value = 0.69, Spearman’s rank correlation −0.27, 95% confidence interval [−0.15, 0.14]). Additionally, based on leave-one-out cross-validation, age at which biopsy was taken was not included in the final ordinal regression model.

### 2.2. Targeted Next Generation Sequencing of Candidate Genes

All samples were successfully sequenced and had a call rate >80%. Median coverage was 69x (SD 15) and complexity 36 (SD 4). Seven genes, *GFER-201*, *MGST2*, *FOXA1*, *COX17*, *ACTA2*, *LTF* and *COMMD10*, were not covered more than 6 times for the complete gene, and thus were not reliably covered. However, variants identified in these genes were still included in the statistical analysis. The sample which was included twice in the NGS showed 99% consistent genotyping results for the identified variants. Genotypes found for the *ATP7B*:c.4373G>A and *ATP7A:980C>T* mutations were respectively 96% and 97% consistent with earlier obtained genotyping results.

A total of 59 variants were identified in addition to the previously identified variants in *ATP7A* and *ATP7B*. Of these variants, six were excluded from statistical analysis because of a call rate <50% and seven were excluded from statistical analysis because of a MAF <3%. Forty-four variants in a total of 25 genes remained (Table S1). Two variants were indels (one splice acceptor variant and one frameshift variant), and the other variants were substitutions (35 missense variants and 10 splice region variants).

**Figure 1:**
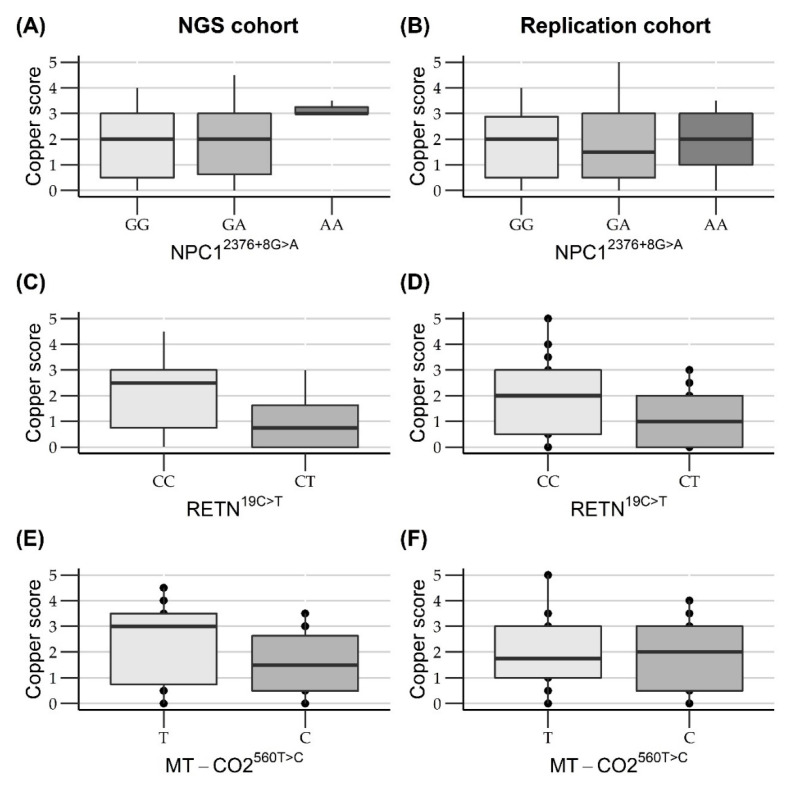
Boxplots of copper score associations between different genotypes in the NGS and replication cohort. (**A**,**B**) Boxplots show a positive significant association between copper score and the non-reference A allele for the variation rs85069010 in *NPC1* in the NGS but not in the replication cohort. (**C**,**D**) Boxplots show a significant negative association between copper score and the non-reference T allele for the variation rs852470997 in *RETN* in both the NGS and replication cohort. (**E**,**F**) Boxplots show a significant negative association between copper score and the non-reference C allele for the variation NC_002008.4:MT.7593T>T in *MT-CO2* in the NGS but not in the replication cohort.

Four variants were significantly or borderline significantly associated with rubeanic acid copper score and were genotyped in a replication cohort of 144 Labrador retrievers. The borderline significant results had an incorporation of 0 in their credibility interval, where one of the credibility limits was closer than 0.01 to zero. To see if a similar trend of association with hepatic copper levels was seen in the replication cohort for these variants, they were also genotyped in the replication cohort. Copper score was positively associated with the non-reference alleles of the non-synonymous nucleotide substitution *COX6B1*.4:c.230G>A (rs852299550) and the splice region variant *NPC1*.3:c.2349+8G>A (rs850609010). A negative association with copper score was found for the non-reference alleles of the non-synonymous nucleotide substitution *RETN*.3:c.19C>T (rs852470997) and the novel non-synonymous nucleotide substitution *MT-CO2*:c.560T>C (NC_002008.4:MT.7593T>C). Sanger DNA sequencing of the *COX6B1* variant rs852299550 showed that there was no variant present at this specific location. The region was sequenced with two different primer sets, and four different primer combinations. For validation animals identified as heterozygous by the NGS were sequenced with all primer sets, and were identified as homozygous wildtype, as illustrated in Figure S1. It was concluded that this variant was an artifact in the NGS and was not actually present in these Labrador retrievers. Therefore, the variant was excluded from further analysis. The other variants were successfully genotyped using either Sanger DNA sequencing or Kompetitive Allelic Specific PCR (KASP) and genotypes found through KASP and Sanger DNA sequencing were consistent with the NGS results. Description of the variants are presented in Table 2 and the outcome of the statistical analysis is presented in Table 3. The relation between the copper score and the genotypes is visualized in Figure 1. Information on the non-significant variants, including the statistics, can be found in Table S1.

### 2.3. Validation of Variants Associated with Hepatic Copper Levels in a Replication Cohort

The non-reference allele frequencies for rs850609010, rs852470997 and NC_002008.4: MT.7593T>C in the replication cohort were 0.26, 0.086 and 0.63 respectively (Table 2). The association with copper score was validated for variant rs852470997 in *RETN*. Statistical results of all the variants are presented in Table 3. The relation between the variants and hepatic copper levels are illustrated in Figure 1.

## 3. Discussion

Copper toxicosis in the Labrador retriever is a complex disease in which both genetic and environmental factors play a role [14]. The disease is characterized by copper accumulation leading to chronic hepatitis and subsequent liver cirrhosis in middle-aged dogs [16]. Previous studies reported an overrepresentation of females in clinical cases [15,16]. In our clinic two-thirds of Labrador retrievers presented with clinical signs of copper associated hepatitis are female, but the mean and median copper levels do not differ between the males and females. Dietary copper uptake can influence the amount of hepatic copper accumulation. Low-copper, high-zinc diets can be part of treatment in dogs with elevated hepatic copper levels, however, treatment effect can vary, with some dogs still accumulating copper while on a copper restricted diet [18]. We previously reported two variants -ATP7B:p.Arg1453Gln and ATP7A:p.Thr327Ile- to be associated with respectively higher, and lower hepatic copper levels in Dutch Labrador retrievers [14]. The variant in *ATP7B* leads to the mis-localization of the protein in the endoplasmic reticulum. The variant in *ATP7A* results in a misfunctioning of the protein, which was demonstrated by copper accumulation and delayed excretion of copper in dermal fibroblasts [14]. In a small study on 10 American Labrador retrievers by Pindar et al. animals homozygous for the *ATP7B* mutation had higher copper levels compared to wildtype and heterozygous individuals [20]. In Dobermanns a hereditary form of copper toxicosis with similarities to the Labrador retriever is described [21]. We reported that the *ATP7B* variant is also associated with elevated copper levels in this breed [22]. In both studies either sample size or non-reference allele frequency was too low to confirm the relation between the *ATP7A* variant and hepatic copper levels [20,21,22].

In Labrador retrievers, the variants in *ATP7A* and *ATP7B* explain only 12.5% of the genetic variation in hepatic copper levels [14]. Therefore, it is expected that variants in other genes play a role in this disease. We performed targeted Next Generation Sequencing of the coding regions of 72 genes involved in copper metabolism or hepatic disease in 95 Labrador retrievers to identify variants that are associated with hepatic copper levels. Associated variants were subsequently genotyped in a replication set of 144 Labrador retrievers. Since, the variants *ATP7A*:c.980C>T and *ATP7B:*c.4358G>A and sex influence hepatic copper, they were taken as covariables in our statistical analysis. Age at the moment of biopsy is potentially associated with hepatic copper levels, with older animals having more time to accumulate copper in their lives, however, within our dataset there was no association found between age at the moment of biopsy and hepatic copper levels, and therefore this was not incorporated as covariable in the statistical analysis. A limitation of the current study was that we could not correct for dietary influence on hepatic copper levels, as no information about dietary copper intake was available for the dogs in this study.

In total we identified 44 variants in 25 genes of which four were associated with hepatic copper levels. These variants were subsequently genotyped in a replication set of 144 Labrador retrievers. A missense variant in *RETN* (c.19C>T; p.Leu7Phe) was found to be negatively associated with hepatic copper score in both the NGS and replication cohort. The non-reference allele frequency in this study was 0.084, which is similar to the non-reference allele frequency of 0.11 found in Polish Labrador retrievers (n = 136) in a study on the relation between polymorphisms in *RETN*, *TNF* and *IL6* and obesity in dogs. No association between obesity and this variant was identified [23]. The variant was also reported in Golden retrievers (n = 36) with a non-reference allele frequency of 0.36, but not in the other breeds included in that study [23].

Resistin -the protein product of the *RETN* gene- was first studied in mice and was classified as an adipokine, since resistin is mainly secreted by white adipocytes and blood cells in mice [24]. In humans, resistin is mainly released by peripheral blood mononuclear cells (PBMCs), macrophages and bone marrow cells and is under study for involvement in various metabolic, inflammatory and autoimmune diseases [25]. Serum resistin levels are associated with non-alcoholic fatty liver disease [26,27,28], degree of liver fibrosis [26,27,29] and degree of liver cirrhosis [30,31] in humans. The protein is expressed in human liver tissue and its expression is upregulated in conditions of chronic injury [32]. Additionally, in mice studies resistin induces hepatic steatosis by diminishing mitochondrial content and downregulating mitochondria, leading to changed mitochondrial morphology and impaired mitochondrial function [33,34]. Similar to humans, resistin is also released by PBMCs in dogs, while release by canine adipocytes could not be identified [35]. Resistin has been studied in dogs in relation to several diseases. No relation was found between canine resistin levels and osteoarthritis [35], pituitary-dependent hyperadrenocorticism [36] and diabetes mellitus [37], but resistin levels were significantly higher in dogs with acute pancreatitis or diabetic ketoacidosis compared to healthy dogs [37,38]. The secretion of resistin by PBMCs and the involvement of resistin in inflammatory disease suggests a pro-inflammatory role of resistin in dogs. No studies are currently available on the relation between hepatic disease and resistin levels in dogs, additionally, to the authors knowledge, no studies are yet performed on the role of resistin in copper accumulation disorders. In this study, we were only able to study the association between this variant and hepatic copper levels in heterozygous and homozygous wildtype individuals due to the low non-reference allele frequency. We did not identify any individuals that were homozygous for the non-reference allele. The allele was in Hardy-Weinberg equilibrium. Further validation of our finding is necessary to elucidate its role in copper toxicosis in dogs, including studying this variant in a larger group of Labrador retrievers and assessing *RETN* expression levels in liver tissue and functional studies.

Two of the genes we selected for sequencing were previously reported to be associated with copper toxicosis in the Bedlington terrier. The first is *COMMD1,* in which the deletion of exon 2 leads to an autosomal recessive, severe form of copper toxicosis [39,40]. Our research group studied the coding and non-coding regions of *COMMD1* in Dobermanns and Labrador retrievers for variants associated with hepatic copper levels [19]. The exon 2 deletion described in Bedlington terriers was not found in the Labrador retrievers and Dobermans in this study. Variants found in *COMMD1* were all in non-coding regions and were not associated with hepatic copper levels [19]. Our data confirmed that there was no association between *COMMD1* variants and hepatic copper levels in Labrador retrievers. Additionally, we sequenced the whole *COMMD* gene family, and found no association between variants and hepatic copper levels. In the Bedlington terrier breed there are dogs with copper toxicosis while being homozygous wildtype or heterozygous for the exon 2 deletion allele in *COMMD1* [41]. In this group of Bedlington terriers the non-reference allele of splice-region variant rs852460740 in *ABCA12* was found associated with increased hepatic copper [41]. The described variant was identified in our NGS cohort with a non-reference allele frequency of 0.72. There was no association between this variant and hepatic copper levels in the NGS cohort (95% credibility interval; −0.615; 0.109). Haywood et al. reported that they found no effect of this variant on the length of the mRNA of ABCA12 and discussed that the variant might not be the actual causal mutation [41], which could explain why no association between copper levels and this variant was found in our study.

The aim of this study was to identify clinically relevant variants that are associated with hepatic copper levels in Labrador retrievers. The coding regions and intron-exon boundaries of 72 genes were sequenced. With this study design, mutations located in promotor regions or non-coding regions will not be detected, however could still influence gene function. From the 72 included genes, seven were insufficiently covered, including the copper chaperone *COX17*, we can’t exclude that variants influencing hepatic copper levels are present in the coding regions of these genes. The variants that we did identify in these genes were not associated with hepatic copper levels. Hepatic copper levels in this study were quantified using a histological scoring system instead of quantitative copper levels, because these were not available for all study subjects. However we found a high correlation between this scoring system and quantitative copper levels of 0.82 (0.76–0.86), which is similar to what Wu et al. found in their study [22]. The sample size of our NGS cohort (n = 95) means that, in agreement with the aim of this study, clinically relevant effects could be detected, however the power of the study was too limited to detect minor effects of polymorphisms on hepatic copper levels.

In conclusion, we found that the missense variant (c.19C>T; p.Leu7Phe) in *RETN* is associated with hepatic copper levels in Labrador retrievers. Further studies, also involving in vitro studies to the effect of the polymorphism on *RETN* expression and resistin functionality, are needed to validate the role in copper metabolism disorders.

## 4. Materials and Methods

### 4.1. Dogs

Research subjects were selected from patients at the Faculty of Veterinary Medicine, Utrecht University or samples sent in from veterinary specialist clinics in the Netherlands between 1999 and 2019. Labrador retrievers of which DNA was stored in our DNA databank, a liver biopsy was available, were not treated with chelation therapy before the liver biopsy and of whom informed consent of owners was acquired, could be included in the study. Additionally, medical history was checked for diseases other than copper associated hepatitis that could influence hepatic copper levels and inclusion of these dogs were determined on a case-by-case basis. Of the dogs included in the study, five Labrador retrievers had neoplasia of the liver, of all these dogs adjacent normal liver parenchyma was available to evaluate hepatic copper levels, one dog presented with cholestasis due to a gallbladder mucocele, but had normal hepatic copper levels. The dogs were either referred to the clinic at Utrecht University because of clinical signs of hepatic disease or increased hepatic parameters in the blood. Records of Labrador retrievers without clinical signs and normal hepatic blood parameters were also available. These animals were investigated because of an ongoing screening program for copper toxicosis at Utrecht University and were examined either because owners intended to breed them or because they had a family history of copper toxicosis. In total 95 dogs were selected for targeted Next Generation sequencing and 144 were selected for inclusion in a replication cohort.

Liver biopsies were obtained for diagnostic purposes. Samples obtained before 2007 were obtained using the Menghini technique other samples were obtained ultrasound guided with a Tru-cut device and 14 gauge needle, or during laparoscopy or laparotomy [42]. From seven dogs liver tissue was acquired post-mortem, the dogs either died because of liver disease (n = 5) or from an unrelated illness (hemangiosarcoma and volvulus of jejunum and ileum). Liver biopsies were embedded in paraffin and 4 µm thick sections, mounted on glass slides, were stained with hematoxylin and eosin for routine evaluation, with rubeanic acid [43] to semi-quantitatively score copper and with the protocol of Gordon and Sweets for fibrosis evaluation [44]. Samples were evaluated and graded according to the World Small Animal Veterinary Association standards, 204 biopsies were scored by board-certified pathologist T.S.G.A.M. van der Ingh and 35 by board-certified pathologist G. Grinwis, who are both specialized in liver pathology [45]. Hepatic copper accumulation was semi-quantitatively scored on a scale from 0 to 5 as previously described [46]. In 176 dogs, additional quantitative copper (CuQ) measured by instrumental neutron activation was available [47]. DNA from the DNA databank of Utrecht University was used for genotyping and sequencing. The biological material used for this study was leftover material of samples obtained for purposes of veterinary care and do not fall under the Dutch rules and regulations of animal testing, therefore no application for a license was made.

### 4.2. Genotyping Variants ATP7A:980C>T and ATP7B:c.4358G>A

Genotyping the *ATP7A:980C>T* (XM_005641519.1) variant and the *ATP7B:c.4358G>A* (XM_005633826.1) variant as described in Fieten et al., 2016 [14], was done with KASP as previously described by Wu et al., 2019 [22].

### 4.3. Targeted Next Generation Sequencing of Candidate Genes

#### 4.3.1. Candidate Gene Selection

Candidate genes that potentially play a role as copper toxicosis disease modifier were selected based on a literature review. Genes with a known role in copper homeostasis were selected, as well as closely related genes or genes described with a potential role in copper metabolism or the etiology of copper metabolism disorders. Additionally, the full family of COMMD genes was included, as well as genes involved in the copper metabolism of the mitochondrion and genes involved in the integration of copper in the mitochondrial respiratory chain. While previously studied in Labrador Retrievers, *ATP7A* and *ATP7B* were included, as part of quality control to compare genotypes of *ATP7A:980C>T* and *ATP7B:c.4358G>A* previously obtained through KASP with the results of the NGS. In total, we included 74 genes in this study of which an overview can be found in Table S2.

#### 4.3.2. Sample Preparation, Sequencing and Mapping

An in-solution enrichment array was designed based on the genomic sequence of Canis Familiaris Build 3.1.72 (CanFam 3.1) [48], and ordered from Agilent Technologies (Santa Clara, CA, USA) as a SureSelect enrichment kit. In the design, all exons and intron-exon boundaries of the selected genes were included, with a total design size of 0.19 Mb. Two μg of genomic DNA per sample was purified using the Qiaquick PCR Purification Kit (Qiagen, Venlo, The Netherlands). DNA was subsequently sheared using a 96 microTube-50 AFA Fiber Plate (Covaris, Woburn, MA, USA). Barcoded fragment libraries were generated from the sheared DNA as previously described [49]. Enriched libraries were sequenced on a SOLiD 5500XL instrument according to the manufacturer’s protocol (Thermo Fischer Scientific, Waltham, MA, USA). Reads were mapped on the dog genome (CanFam 3.1.) using a Burrows-Wheeler Aligner (BWA) algorithm [50] with the following parameters (-c –l 25 -k 2 -n 10) and variant calling was done using a custom pipeline identifying variants with at least 10x coverage, a 15% allele frequency as well as support from independent reads (>3).

#### 4.3.3. Quality Control and Filtering

To be included in the analysis, samples had to have a minimum call rate of 80%. Minimum depth was set at 6x and if the non-reference allele was called between 25% and 75% of the reads, the animal was classified as heterozygous. These boundaries and the quality of the NGS were assessed through the variants *ATP7A:980C>T* and *ATP7B:c.4358G>A*, which were also genotyped with KASP for all animals. Variants with a call rate below 50% and/or a MAF below 3% were excluded from the analysis. Variants were annotated with their SIFT [51,52], Polyphen-2 [53] and GERP-score [54]. The integrated genomic viewer (IGV) version 2.8.3 was used to assess regions with low coverage.

#### 4.3.4. Statistical Analysis

All analyses were applied in R version 4.0.2 (R Core Team, 2020). Genotype data was analyzed using R package brms version 2.13.0. The brms package performs Bayesian estimation using the Stan programming language [55]. An ordinal regression model with cumulative link function (probit) was used to assess the association between copper score and a specific variant, with (full model) and without (crude analyses) sex, *ATP7A* and *ATP7B* genotype as covariables. With relation to hepatic copper levels the assumption was made that the underlying variable for the copper score was continuous. Since age of the dog at moment of biopsy could be a confounder, the necessity to include this factor into the model was assessed. Firstly, a univariate analysis was performed using a Spearman’s rank correlation analysis. Additionally ordinal regression models were fitted with and without age at biopsy and leave one out cross validation was used to determine whether this factor should be included in the model. This analysis was done using all 239 dogs included in the study. To assess the relation between quantitative copper levels and the copper score, a Spearman’s rank correlation analyses was performed. Since only two animals had a copper score of 5, these were recoded as levels 4 before analysis, to avoid problematic small group sizes. Genotypes were modeled additively. For the X-chromosomal *ATP7A* variant, hemizygous dogs were scored as 0 (CY) or 2 (TY). The results of the models were presented as estimates with 95% credibility intervals on the probit sale. A variant was assessed as significantly associated with hepatic copper levels if the credibility interval of the full model analysis did not contain 0. Statistical analysis for the variants genotyped in the replication cohort was identical to the NGS cohort. To evaluate if there were any significant differences between the baseline characteristics of the two cohorts Pearson’s Chi-squared test was used for the non-reference allele frequencies of *ATP7A* and *ATP7B* genotypes and female to male ratio. The previous described ordinal regression model was used to evaluate differences in copper levels between the cohorts.

### 4.4. Genotyping of Variants in a Replication Cohort

Variants found significantly associated with hepatic copper quantity were validated in a replication cohort of 144 dogs through Sanger DNA sequencing or KASP. Additionally, all 95 dogs included in the NGS cohort were also genotyped to validate the genotypes found in the NGS. Four variants were selected for genotyping in the replication cohort. Variants NC_006583.3:g.116961020C>T, NC_006589.3.g.64792857G>A, NC_006602.3.g.52434480C>T were genotyped using Sanger DNA sequencing and variant NC_002008.4:MT.7593T>C was genotyped using KASP.

The primers presented in Table 4 were used to amplify the selected regions. For variant NC_006583.3:g.116961020C>T, two primer sets and four different primer combinations were used, since initial results were different from the NGS results. The PCR amplification was performed using 1× Platinum^®^ PCR Buffer, dNTPs 0.5 µM each, 2 mM MgCl_2_, 0.5 mM of each forward and reverse primer, 1 Unit Platinum^®^ Taq DNA polymerase (Invitrogen, Carlsbad, CA, USA) and about 50 ng of gDNA in a reaction volume of 15 µl. The thermal cycling protocol consisted of a 5 min denaturation step at 95 °C, 35 cycles of 30 s at 95 °C, 30 s at 55 °C and 30 s at 72 °C. After this a final elongation step at 72 °C for 10 min was performed. The PCR products were incubated with 2 units of Exonuclease I (New England Biolabs, Ipswich, MA, USA) in a thermal cycler at 37 °C for 45 min and the enzyme was inactivated at 75 °C for 15 min. DNA sequence reactions were performed using BigDye v3.1 according to the manufacturer’s (Applied Biosystems, Foster City, CA, USA) protocol. All amplifications were performed on an ABI 9700 Thermal Cycler (Applied Biosystems). The DNA sequencing reactions were purified with Sephadex G50 (GE-Healthcare, Chicago, IL, USA) and loaded onto an ABI3130XL. The obtained sequences were analyzed in Lasergene (version 16 DNASTAR).

One variant was genotyped through a KASP. The procedure was performed in compliance with the manufacturer’s instructions (LGC Genomics, Teddington, UK). A CFX384 Touch^TM^ Real Time PCR detection system (Bio-Rad, Hercules, CA, USA) was used for fluorescence signal detections and results were analyzed by Bio-Rad CFX manager 3.0 (Bio-Rad).

## Figures and Tables

**Table 1:** Characteristics of the NGS and replication cohort.

Cohort		Group Size	Copper Score	Age	Non-ref. Allele Freq.^1^ *ATP7A*:980C>T	Non-Ref. Allele Freq. ATP7B:c.4373G>A
NGS	Females	65	3 (0–5)	5.6 (0.9–13.5)	0.24	0.35
	Males	30	1 (0–4)	5.3 (1.4–9.0)	0.33	0.32
	Total	95	2 (0–5)	5.3 (0.9–13.5)	0.29	0.34
Replication	Females	97	2 (0–5)	6.7 (0.9–12.7)	0.28	0.30
	Males	47	2 (0–4)	7.3 (1.5–12.5)	0.26	0.36
	Total	144	2 (0–5)	7.0 (0.9–12.7)	0.28	0.32

Characteristics of the NGS and replication cohort are presented for males and females separately and additionally for the complete group. Age at liver biopsy and the copper score are described with median and range. ^1^ Non-reference allele frequency.

**Table 2:** Description and statistics of the gene variants associated with copper score.

Gene	Description	Non-ref. Allele Freq.^1^ NGS/Replication Cohort	Variant Type	Gene Function
*NPC1*	NC_006589.3.g.64792857G>A (rs850609010)	*0.22/0.26*	splice region	Intracellular cholesterol transporter
*RETN*	NC_006602.3.g.52434480C>T (rs852470997)	*0.08/0.09*	Missense Leu7Phe	Pro-inflammatory adipokine associated with hepatic fibrosis and cirrhosis
*MT-CO2*	NC_002008.4:MT.7593T>C (novel)	*0.63/0.63*	Missense Met187Thr	Subunit of Cytochrome C Oxidase

Description of the variants associated with hepatic copper score. ^1^ Non-reference allele frequency.

**Table 3:** Effect estimates and credibility intervals of the association between copper score and variants for the NGS and replication cohort.

Gene	Variant	NGS Cohort; Crude Analysis	NGS Cohort; Full Model Analysis	Replication Cohort; Crude Analysis	Replication Cohort Full Model Analysis
*NPC1*	rs850609010	0.307 (−0.074; 0.689)	0.384 (−0.0029; 0.775)	0.036 (−0.244; 0.318)	0.039 (−0.240; 0.320)
*RETN*	rs852470997	**−0.731 (−1.315; −0.148)**	**−0.662 (−1.253; −0.073)**	−0.478 (−0.977; 0.018)	−0.527 (−1.033; −0.016)
*MT-C*	NC_002008.4: MT.7593T>C	**−0.267 (−0.487; −0.046)**	−0.226 (−0.456; 0.0037)	−0.081 (−0.269; 0.108)	−0.278 (−0.278; 0.102)

Association was analyzed using an ordinal regression with a proportional odds function on a probit scale, the non-reference allele was assessed additively. Crude analysis was a model only including copper score and genotype of studied variant. The full model is corrected for sex, *ATP7A c.980C>T* and *ATP7B* c.435G>A genotype. Estimates and 95% credibility interval are presented. Significant results are presented in bold.

**Table 4:** Oligonucleotides used for genotyping the replication cohort.

Variant	Primer	Sequence
rs852299550	F1	5′-AGTTAAGCCATGATTCAGGATCCC-3′
rs852299550	R1	5′-GGGTCACTGCTGATTCCCTC-3′
rs852299550	F2	5′-CTGGGAGAAGGATGGAGGAC-3′
rs852299550	R2	5′-CCGACTAAACAGGTAGGCAGAG-3′
rs850609010	F	5′-AGGAGCCTTGTCACAGATGC-3′
rs850609010	R	5′-CCACCTTCAGAGCCTGTGAG-3′
rs852470997	F	5′-CATCCAGAGGGTAAGTGACAGC-3′
rs852470997	R	5′-CTGACATTGGAAGCGAGGGT-3′
NC_002008.4:MT.7593T>C	A1	5′-GAAGGTGACCAAGTTCATGCTGCCATAGTACAGTCCTGGTCGTA-3′
NC_002008.4:MT.7593T>C	A2	5′-GAAGGTCGGAGTCAACGGATTCCATAGTACAGTCCTGGTCGTG-3′
NC_002008.4:MT.7593T>C	R	5′-GGACGACTAAACCAAACCACCCTTA-3′

Oligonucleotides used for genotyping in the replication cohort. F stands for forward primer and R for reverse primer. A1 and A2 are the allele specific primers used in the KASP.

## Supplementary Materials

Supplementary Materials can be found at https://www.mdpi.com/2075-1729/10/11/266/s1. Table S1: Identified polymorphisms, characteristics and statistical analysis. Table S2: Gene selection for targeted NGS. Figure S1: Illustration of Sanger DNA sequencing results for variant rs852299550 of a sample that was called heterozygous in the NGS.

## Abbreviations

BCS	Body Condition Score
Cu	Copper
CuQ	Quantitative copper
KASP	Kompetitive Allele Specific PCR
NGS	Next Generation Sequencing
PBMC	Peripheral blood mononuclear cells
RA	Rubeanic Acid
ROS	Reactive Oxygen Species
TGN	*trans*-Golgi Network
