# Supplementary material for: Investigation of Genetic Modifiers of Copper Toxicosis in Labrador Retrievers
Source: Life (Basel). 2020 Oct 31;10(11):266. doi: 10.3390/life10110266 (PMC7693796; doi:10.3390/life10110266)
Supplement: Supplementary file 1 [file life-10-00266-s001.zip › supp-life-985718/Supplemental Figure 1.docx]

Investigation of Genetic Modifiers of Copper Toxicosis in Labrador Retrievers

**Supplementary Material**


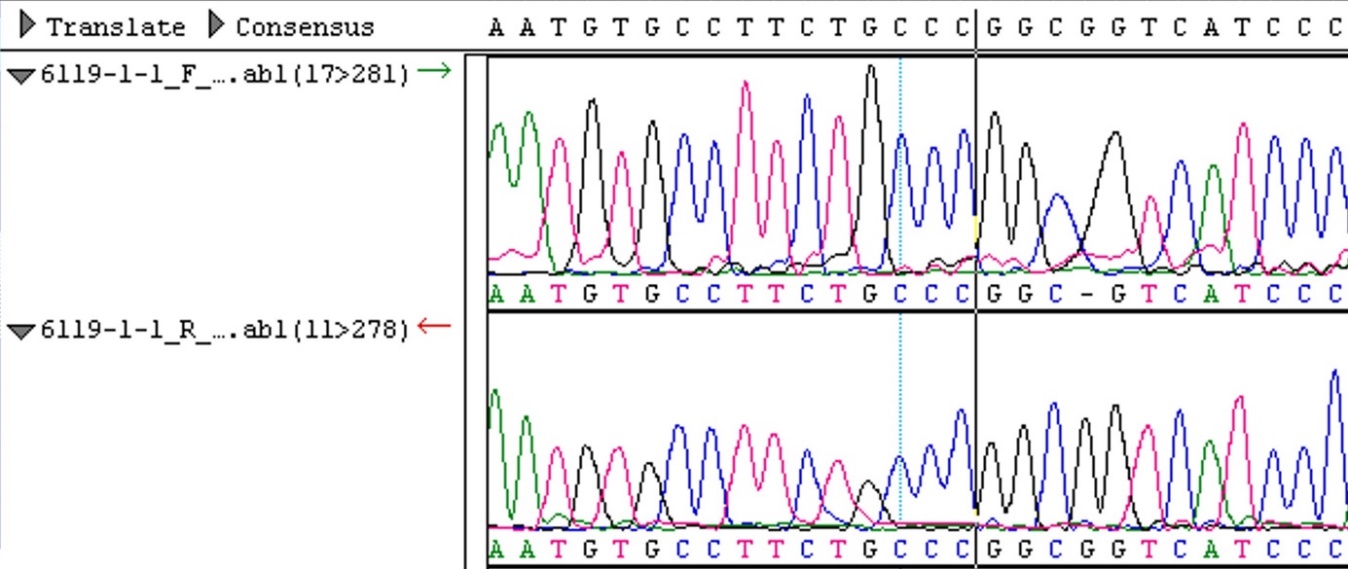


**Figure S1.** Illustration of Sanger DNA sequencing results for *COX6B1*^c.230G>A^ variant rs852299550 of a sample that was called heterozygous in the NGS. Black line marks expected position of the presumed variant.
